# Tetrabromobisphenol A and Diclazuril Evoke Tissue-Specific Changes of Thyroid Hormone Signaling in Male Thyroid Hormone Action Indicator Mice

**DOI:** 10.3390/ijms232314782

**Published:** 2022-11-26

**Authors:** Richárd Sinkó, Kristóf Rada, Anna Kollár, Petra Mohácsik, Miklós Tenk, Csaba Fekete, Balázs Gereben

**Affiliations:** 1 Laboratory of Molecular Cell Metabolism, Institute of Experimental Medicine, H-1083 Budapest, Hungary; 2 János Szentágothai PhD School of Neurosciences, Semmelweis University, H-1085 Budapest, Hungary; 3 Department of Microbiology and Infectious Diseases, University of Veterinary Medicine, H-1143 Budapest, Hungary; 4 Laboratory of Integrative Neuroendocrinology, Institute of Experimental Medicine, H-1083 Budapest, Hungary

**Keywords:** TBBPA, diclazuril, endocrine disruption, tissue-specific thyroid hormone action, thyroid hormone action indicator mouse

## Abstract

Thyroid hormone (TH) signaling is a prerequisite of normal tissue function. Environmental pollutants with the potential to disrupt endocrine functions represent an emerging threat to human health and agricultural production. We used our Thyroid Hormone Action Indicator (THAI) mouse model to study the effects of tetrabromobisphenol A (TBBPA; 150 mg/bwkg/day orally for 6 days) and diclazuril (10.0 mg/bwkg/day orally for 5 days), a known and a potential hormone disruptor, respectively, on local TH economy. Tissue-specific changes of TH action were assessed in 90-day-old THAI mice by measuring the expression of a TH-responsive luciferase reporter in tissue samples and by in vivo imaging (14-day-long treatment accompanied with imaging on day 7, 14 and 21 from the first day of treatment) in live THAI mice. This was followed by promoter assays to elucidate the mechanism of the observed effects. TBBPA and diclazuril impacted TH action differently and tissue-specifically. TBBPA disrupted TH signaling in the bone and small intestine and impaired the global TH economy by decreasing the circulating free T4 levels. In the promoter assays, TBBPA showed a direct stimulatory effect on the *hdio3* promoter, indicating a potential mechanism for silencing TH action. In contrast, diclazuril acted as a stimulator of TH action in the liver, skeletal muscle and brown adipose tissue without affecting the Hypothalamo-Pituitary-Thyroid axis. Our data demonstrate distinct and tissue-specific effects of TBBPA and diclazuril on local TH action and prove that the THAI mouse is a novel mammalian model to identify TH disruptors and their tissue-specific effects.

## 1. Introduction

Thyroid hormone (TH) signaling is a well-known, fundamental regulator of cellular functions. Physiological levels of TH action represent a prerequisite of normal tissue function during development and adulthood [1]. Tissue TH action is regulated by a complex machinery that allows the generation and maintenance of tissue-specific signatures of TH action that can be independent of the relatively stable circulating serum TH levels [2,3,4,5,6].

Environmental pollutants with endocrine disruptor activity represent a growing concern, since these molecules reach the food chain via water and agricultural production and consequently can seriously impact human and animal health [7,8,9]. Despite intense efforts and progress made in the screening of potential endocrine disruptor activities, our knowledge is still limited concerning the impact of these molecules on tissue-specific TH action. This is also associated with the limitations of the available experimental models [10,11,12]. While in recent years the number of novel test systems has appeared to be growing, a common aspect of most in vivo models is the use of endogenous genes as markers of TH action. These genes are simultaneously regulated by numerous signaling systems, with potential interference between direct and indirect effects of the tested disruptor on TH economy [13,14]. Therefore, the assessment of endocrine disruptor activity in mammalian tissues remains a major challenge.

We generated the THAI transgenic mouse model, which has been proven suitable to selectively assess tissue-specific TH action in an unbiased manner using a TH-responsive luciferase reporter system while all members of the local TH signaling machinery remain intact [15,16,17]. We used this model to assess the endocrine disruptor activity of two compounds, tetrabromobisphenol A (TBBPA) and diclazuril.

Tetrabromobisphenol A (TBBPA) is the most common flame retardant used for the production of printed electronic circuit boards, various plastic products and textiles [18,19]. It is discharged into the environment during manufacturing, use and disposal of electrical equipment, which results in the contamination of air, water, soil, sediments and sewage sludge [20,21]. TBBPA was found to be a TH disrupting agent by studying *Rana* and *Xenopus* metamorphosis [10,22].

Alarmingly, TBBPA was also detected in aquatic food samples at concentrations as high as 207.3 ng/g lipid weight, further increasing human exposure [23]. Consequently, TBBPA was also found in human tissues, milk and serum, and its concentrations reached 37 ng/g in breast milk and 649 ng/g in umbilical cord serum [8,24,25]. In addition, TBBPA showed neurotoxic, nephrotoxic and hepatotoxic effects and also impacted reproductive health in various animal models [10,26,27]. In human studies, it has been shown to affect the endocrine and immune systems especially during development and pregnancy [12,28,29]. The acute toxicity indicated by the LD50 was determined to be between 5 and 10 g/bwkg after a single oral dose in mice, rats and rabbits [30].

Diclazuril is widely used as an antiprotozoal agent and acts by targeting the chlorophyll a-D1 complex. It is used to prevent and treat coccidiosis in multiple species and is also applied against equine protozoal myeloencephalitis, and to a lesser extent, toxoplasmosis and neosporosis [31,32]. Its oral or subcutaneous dosages up to 5000 mg/bwkg caused no mortality in mice and rats [33].

Coccidiosis poses an especially significant health risk in poultry, with significant economic consequences. As chickens are often treated with medicated food containing diclazuril, human exposure is a realistic scenario. Thus, the effect of diclazuril on the human TH economy needs to be further investigated [34]. Diclazuril is considered to be safe against toxoplasmosis during pregnancy in a mouse model [35]. However, continuous exposure leads to stable plasma levels, which raises human concerns and calls for further studies. Furthermore, its potential to disrupt hormonal signaling is poorly documented, but its ability to bind androgen receptors was shown, along with data of its potential to antagonize TH receptors in a high-throughput cell-based reporter gene assay [36,37].

Our data obtained in the THAI mouse demonstrate that TBBPA and diclazuril exert a tissue-specific impact on mammalian TH action detectable in living animals and in isolated tissue samples. The obtained data demonstrated the tissue-specific effects of TBBPA and diclazuril on local and global TH economy. They also proved that the THAI mouse provides a selective in vivo tissue-specific mammalian model to screen the potential of compounds to disrupt TH signaling.

## 2. Results

### 2.1. Tissue-Specific Effects of TBBPA and Diclazuril on Peripheral TH Action

To assess the effect of TBBPA and diclazuril on TH action in different peripheral tissues, we used our THAI mouse as an animal model [15]. TH action was assessed in tissue samples by measuring the mRNA level of the TH-responsive luciferase reporter system in dissected tissues with Taqman qPCR. We administered 150 mg/bwkg/day of TBBPA for 6 days and 10.0 mg/bwkg/day of diclazuril for 5 days by oral gavage.

**Figure 1:**
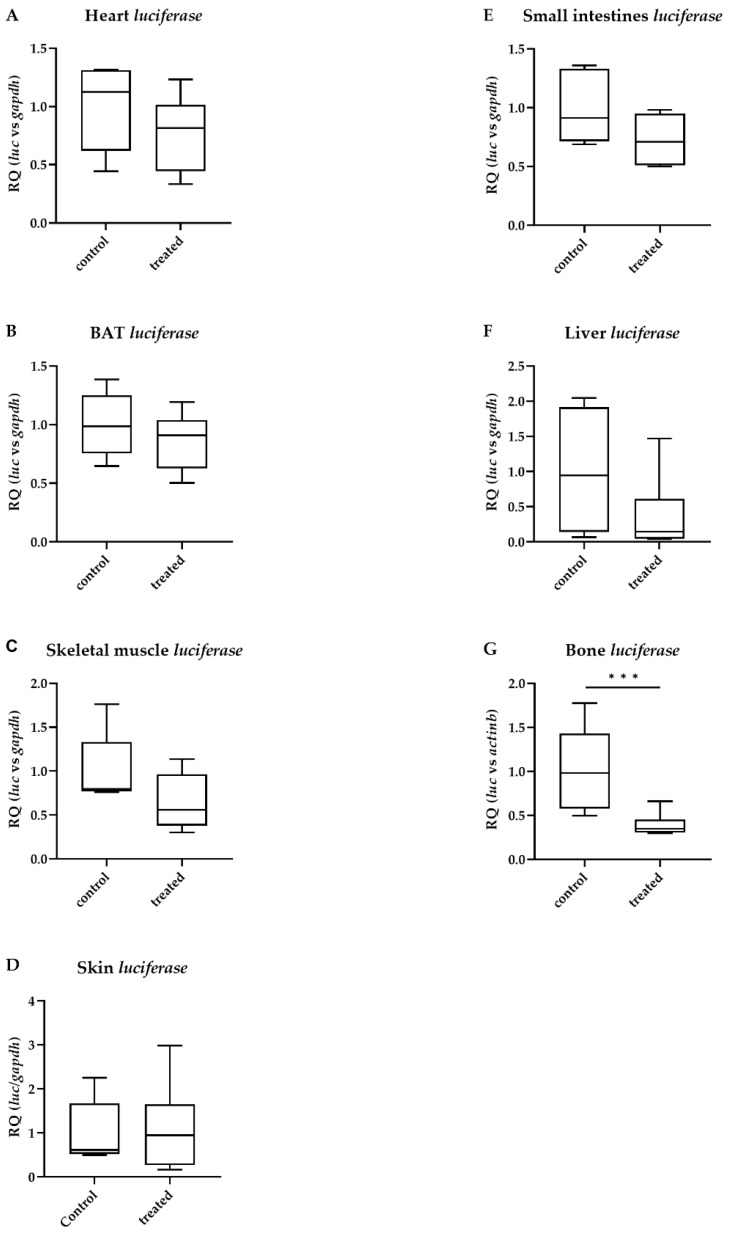
Peripheral thyroid hormone action after tetrabromobisphenol A (TBBPA) treatment. Thyroid hormone action quantified with *luciferase* mRNA levels in male THAI mice after six days of oral administration of 150 mg/bwkg/day of TBBPA in corn oil containing 2% Et-OH; (**A**) heart; (**B**) brown adipose tissue; (**C**) skeletal muscle; (**D**) skin; (**E**) small intestine; (**F**) liver; (**G**) bone. n = 4–6 mice/group; figure shows Tukey Box Plots, α = 0.05; ***: *p* < 0.001.

After the TBBPA treatment, TH action remained unchanged in the heart, interscapular brown adipose tissue (BAT), skeletal muscle, skin, small intestines and liver (Figure 1A–F). In contrast, TH action in the bone was strongly decreased (Figure 1G).

**Figure 2:**
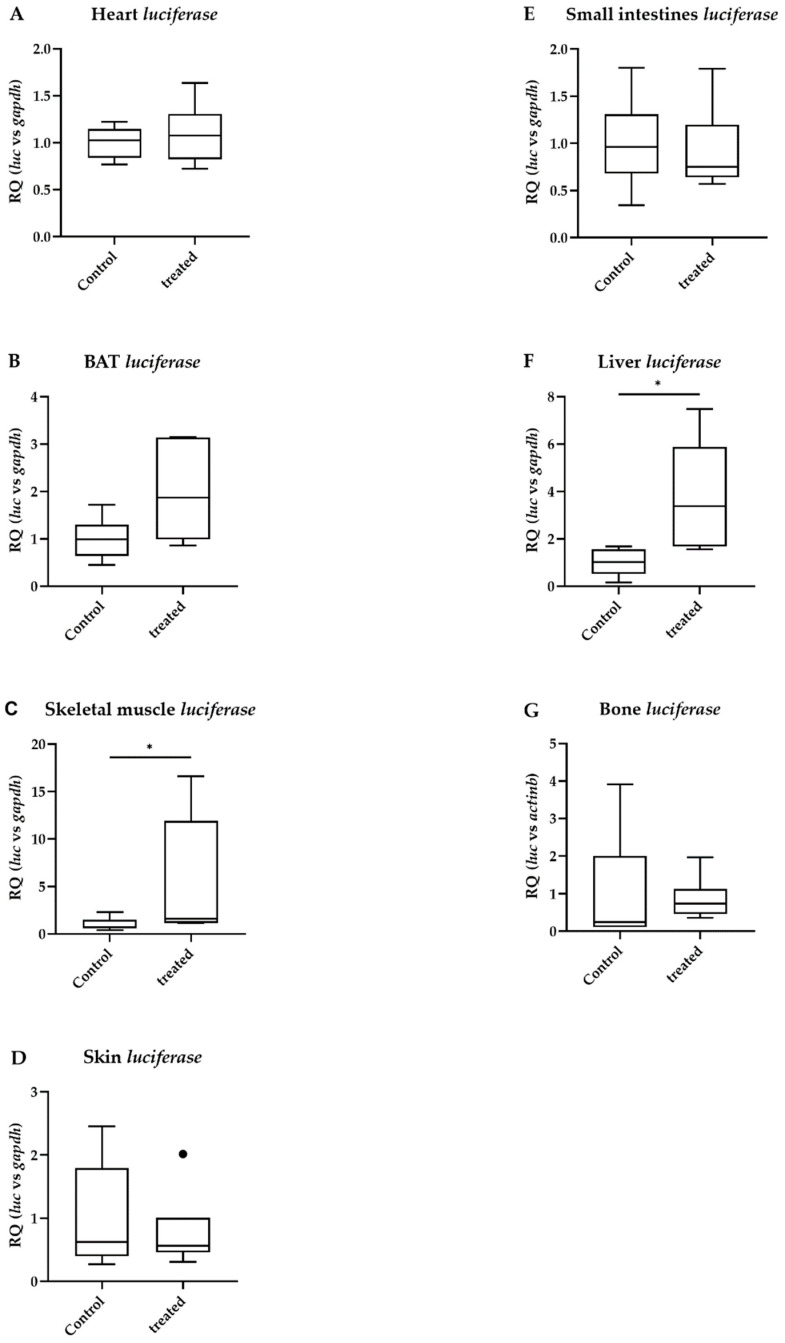
Peripheral thyroid hormone action after diclazuril treatment. Thyroid hormone action quantified with *luciferase* mRNA levels in male THAI mice after five days of oral administration of 10 mg/bwkg/day of diclazuril in saline suspension; (**A**) heart; (**B**) brown adipose tissue; (**C**) skeletal muscle; (**D**): skin; (**E**) small intestine; (**F**) liver; (**G**) bone. n = 5–6/group; figure shows Tukey Box Plots, α = 0.05; *: *p* < 0.05.

Similarly to TBBPA, diclazuril left TH action unchanged in the heart, BAT, skin and small intestine (Figure 2A,B,D,E). However, diclazuril increased TH action in the skeletal muscle and liver (Figure 2C,F) and did not change TH action in the bone (Figure 2G).

### 2.2. Distinct Impacts of TBBPA and Diclazuril on Local TH Action in Live THAI Mice

After assessing the impact of the two compounds in various peripheral tissues, we were interested in whether a less invasive, in vivo method allowing a longer follow-up of the same animal would be adequate to assess trending changes that did not reach significance in tissue homogenates (Figure 1 and Figure 2). Therefore, we subjected the THAI mice to in vivo imaging according to our established protocol that allows the assessment of TH signaling in the small intestine and BAT of the THAI mouse [15]. We measured TH action in these tissues with a longer treatment time than in the previous experiments, using similar doses of the compounds. A 14-day-long treatment was accompanied with imaging on day 7, 14 and 21 from the first day of treatment. The animals were subjected to bioluminescent in vivo imaging before the first treatment, after 1 and 2 weeks of daily treatment and finally after one week from treatment withdrawal. Each animal served as a self-control for its own measurements.

**Figure 3:**
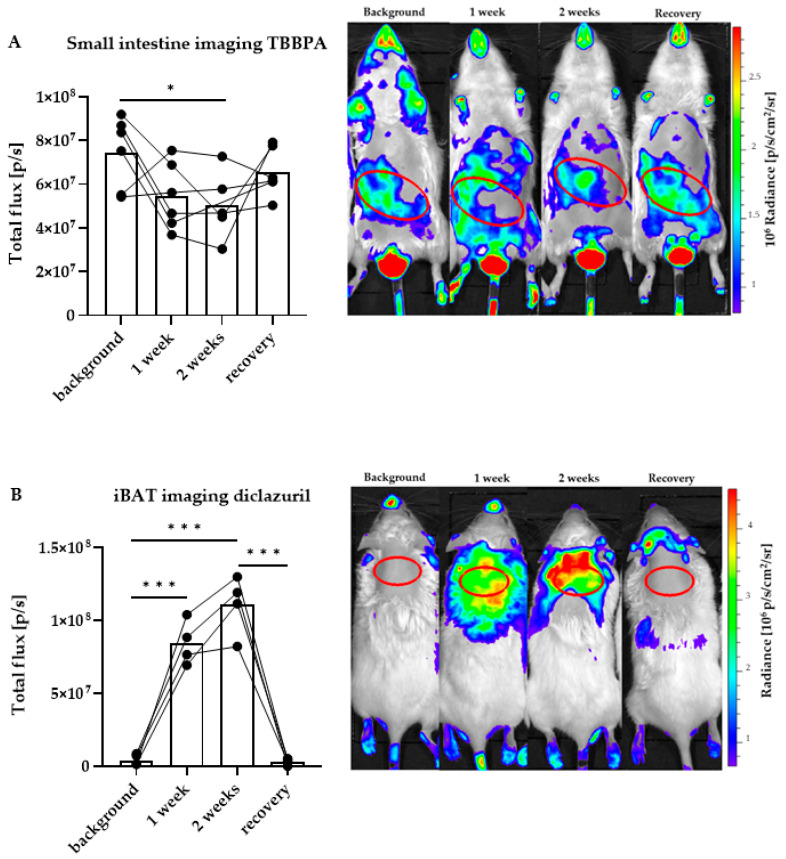
Quantitation of local thyroid hormone action after disruptor treatment with in vivo imaging. Representative images and quantification in male THAI mice treated orally for two weeks with 150 mg/bwkg/day of TBBPA in corn oil containing 2% Et-OH, or 10 mg/bwkg/day of diclazuril as a saline suspension, followed by one week of recovery; (**A**) ventral in vivo imaging of TBBPA treatment; (**B**) dorsal in vivo imaging of diclazuril treatment. n = 4–6 mice/group; figure shows Tukey Box Plots, α = 0.05; *: *p* < 0.05, ***: *p* < 0.001.

In the small intestine, TH action was decreased after 2 weeks of TBBPA treatment and then recovered after the recovery week (Figure 3A). In contrast, diclazuril induced a significantly elevated TH action in BAT that also recovered after 1 week (Figure 3B). The observed changes confirmed the trend obtained in mRNA expression in tissue homogenates (Figure 1E and Figure 2B).

### 2.3. TBBPA Impacts the Hypothalamo–Pituitary–Thyroid (HPT) Axis, While Diclazuril Does Not

To assess whether the observed changes were the consequence of a local impact on TH action or were associated with an altered function of the HPT axis, we measured parameters that hallmark the activity of the HPT axis in animals treated with TBBPA or diclazuril.

**Figure 4:**
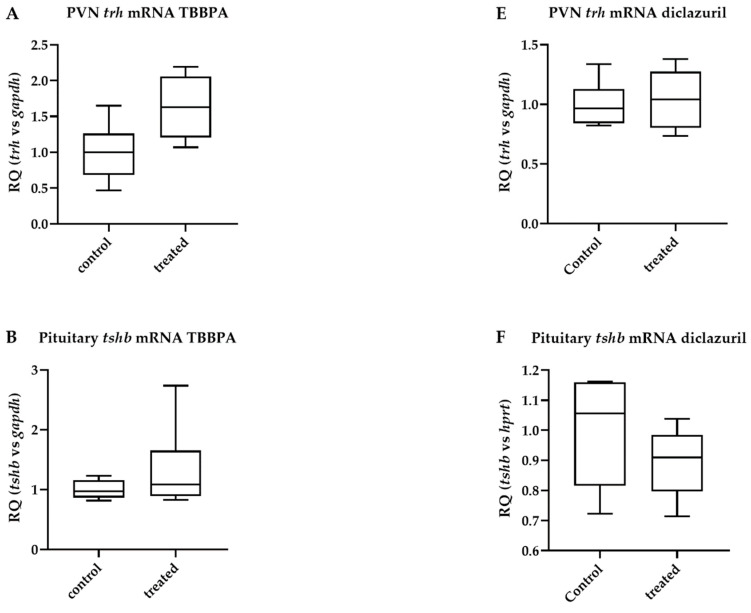

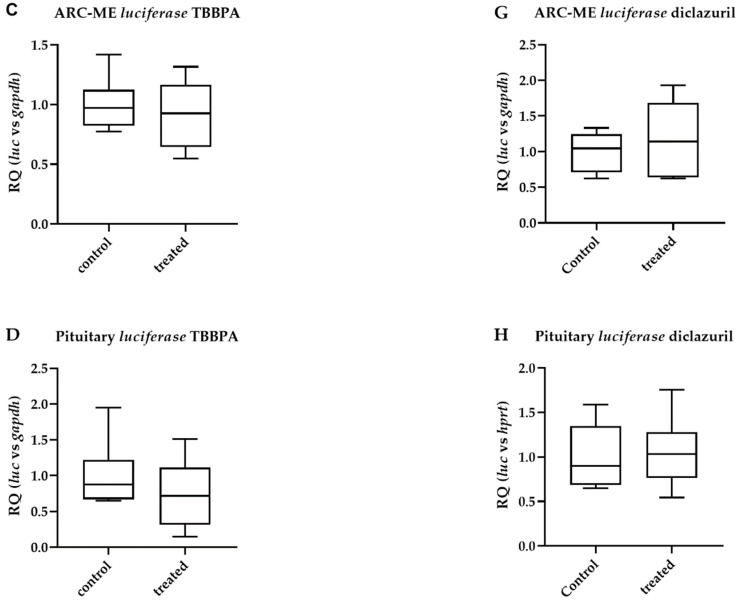
Hypothalamic and pituitary effects of disruptor treatment. Male THAI mice treated orally for 6 days with 150 mg/bwkg/day of TBBPA in corn oil containing 2% Et-OH or for 5 days with 10 mg/bwkg/day of diclazuril as a saline suspension; thyroid hormone action quantified with *luciferase* mRNA levels; (**A**–**D**) TBBPA; (**E**–**H**) diclazuril; (**A**) PVN *trh* mRNA after TBBPA; (**B**) pituitary *tshb* mRNA after TBBPA; (**C**) ARC-ME *luciferase* mRNA after TBBPA; (**D**) pituitary *luciferase* mRNA after TBBPA; (**E**) PVN *trh* mRNA after diclazuril; (**F**) pituitary *tshb* mRNA after diclazuril; (**G**) ARC-ME *luciferase* mRNA after diclazuril; (**H**) pituitary *luciferase* mRNA after diclazuril. n = 4–6 mice/group; figure shows Tukey Box Plots, α = 0.05.

After TBBPA treatment, *trh* expression in microdissected hypothalamic paraventricular nucleus (PVN) samples did not change significantly, despite a trend toward an increase (63% increase, *p* = 0.064). This was accompanied by unaltered *tshb* mRNA levels in the pituitary (Figure 4A,B); in parallel, TH action remained unchanged in the microdissected hypothalamic arcuate nucleus median eminence (ARC-ME) region and in the pituitary (Figure 4C,D). Similar results were obtained with diclazuril (Figure 4E–H), except for the lack of a trend toward an increase in *trh* expression in the PVN.

**Figure 5:**
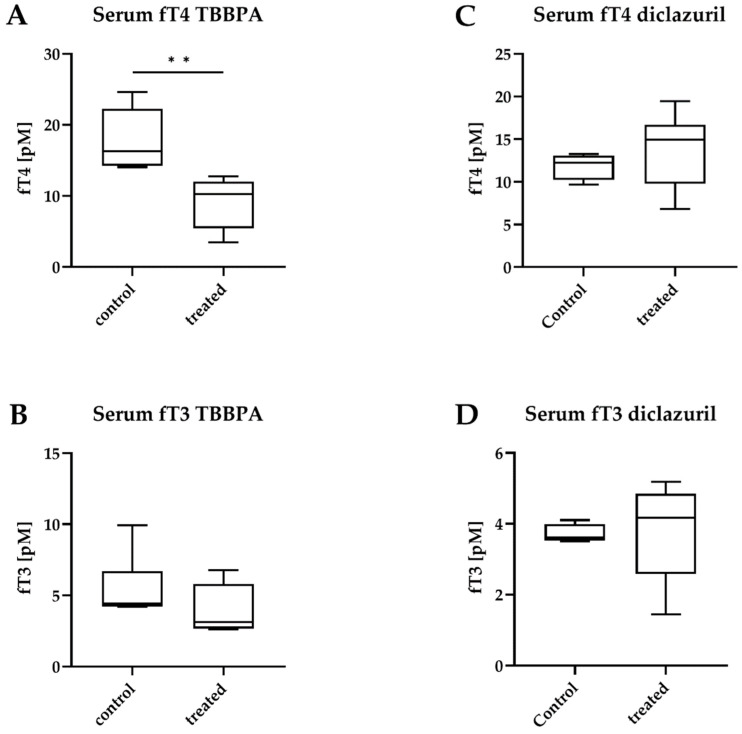
Circulating hormone levels after disruptor treatment. Male THAI mice treated orally for 6 days with 150 mg/bwkg/day of TBBPA in corn oil containing 2% Et-OH or for 5 days with 10 mg/bwkg/day of diclazuril as a saline suspension; (**A**,**B**) TBBPA; (**C**,**D**) diclazuril; (**A**) serum free T4 (fT4) after TBBPA; (**B**) serum free T3 (fT3) after TBBPA; (**C**) serum fT4 after diclazuril; (**D**) serum fT3 after diclazuril. n = 4–6 mice/group; figure shows Tukey Box Plots, α = 0.05; **: *p* < 0.01.

However, despite the unchanged central parameters, massively decreased circulating free T4 (fT4) levels were detected after TBBPA treatment, while free T3 (fT3) remained unchanged (Figure 5A,B). Both fT4 and fT3 remained unchanged after diclazuril treatment (Figure 5C,D).

### 2.4. Diclazuril Does Not Impact the Cerebral Cortex; the Effect of TBBPA on dio3 Expression Is Counterbalanced by Local Mechanisms

**Figure 6:**
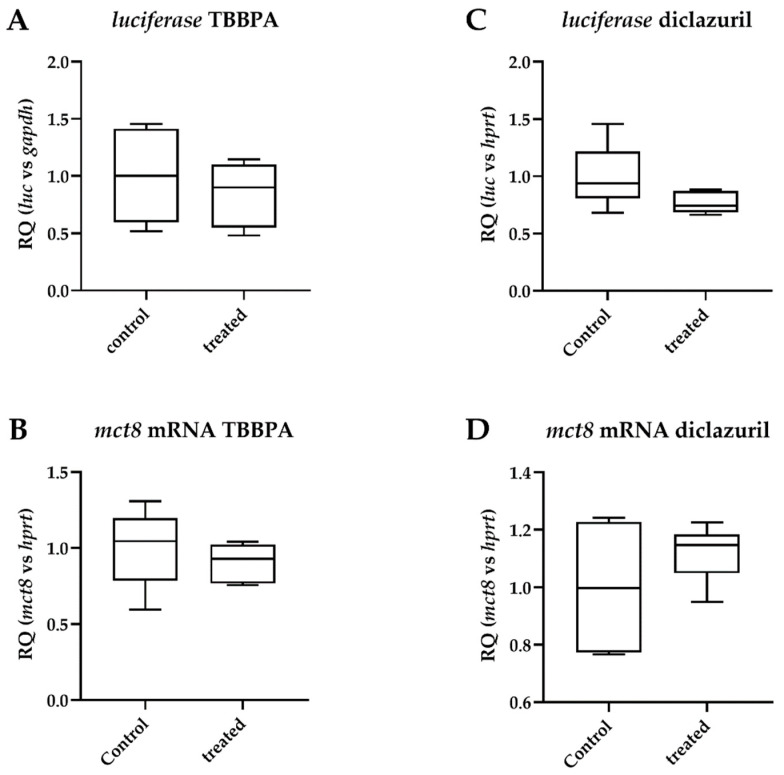
Changes in parameters of thyroid hormone action in the cerebral cortex after disruptor treatment. Male THAI mice treated orally for 6 days with 150 mg/bwkg/day of TBBPA in corn oil containing 2% Et-OH or for 5 days with 10 mg/bwkg/day of diclazuril as a saline suspension; thyroid hormone action quantified with *luciferase* mRNA levels; (**A**,**B**) TBBPA; (**C**,**D**) diclazuril; (**A**) *luciferase* mRNA after TBBPA; (**B**) *mct8* mRNA after TBBPA; (**C**) preamplified *luciferase* mRNA after diclazuril; (**D**) *mct8* mRNA after diclazuril. n = 5–6 mice/group; figure shows Tukey Box Plots, α = 0.05.

Having studied the impact of TBBPA or diclazuril on the HPT axis, we also investigated how the cerebral cortex, a region protected by the blood–brain barrier, was affected by these compounds. Both treatments resulted in unchanged cortical TH action (Figure 6A,C).

Since the cortex is known to be programmed to maintain T3 homeostasis [2], we tried to characterize local regulators of TH action in the cortex under the present treatment. Monocarboxylate transporter 8 (MCT8) is one of the major TH transporters with a critical function in the brain. Neither TBBPA nor diclazuril had an effect on the mRNA levels of *mct8*, suggesting unaltered TH transport (Figure 6B,D).

**Figure 7:**
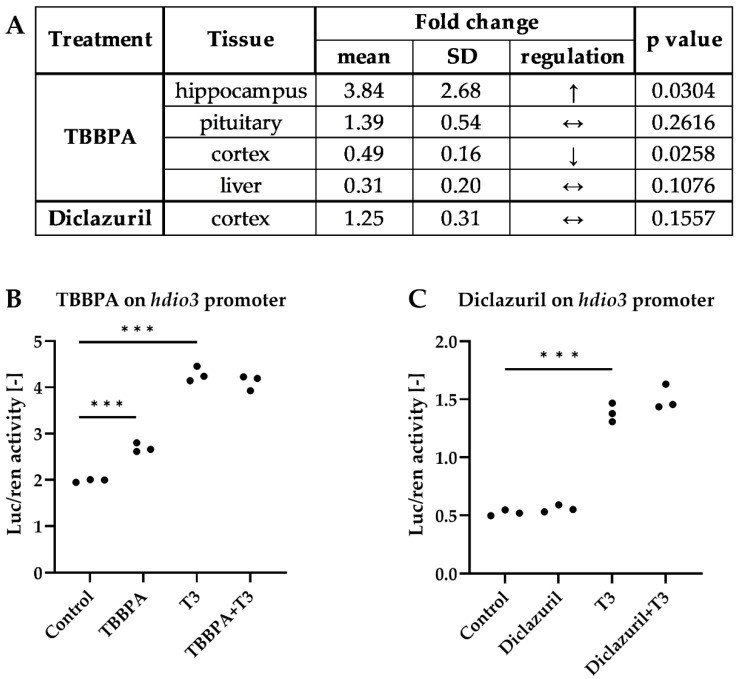
Regulation of *dio3* expression by TBBPA and diclazuril. (**A**) of *dio3* mRNA changes in different tissues of male THAI mice treated orally for 6 days with 150 mg/bwkg/day of TBBPA in corn oil containing 2% Et-OH or for 5 days with 10 mg/bwkg/day of diclazuril as a saline suspension; (**B**,**C**) transfected HEK293T cultures treated with 50 nM T3 and/or 1 μM TBBPA or diclazuril measured with Dual-Luciferase Reporter Assay. n = 4–6 mice/group for A, α = 0.05; ***: *p* < 0.001.

Neuronal TH action is heavily regulated by type 3 deiodinase (D3), the main TH-degrading enzyme [38]. To further characterize the cortical effects of TBBPA or diclazuril, we also studied how *dio3* was impacted. TBBPA exerted a tissue-specific effect on *dio3* expression; it was decreased in the cortex, increased in the hippocampus and remained unchanged in the pituitary and liver (Figure 7A).

To understand whether these were indirect, compensatory changes or direct effects of TBBPA on the *dio3* promoter, we performed promoter assays in HEK293T cell cultures transfected with a luciferase reporter linked to a *dio3* promoter, which enabled the bioluminescent quantitation of promoter activity. TBBPA increased *dio3*-*luciferase* expression in hormone-free medium and did not have an additive effect in the presence of 50 nM T3, that, as expected, also induced reporter expression in the presence of TRβ (Figure 7B). In contrast, diclazuril did not regulate *dio3* promoter activity either in the cerebral cortex or in the in vitro promoter assay (Figure 7A,C). Interestingly, TBBPA is likely able to impact the local TH action in various tissues by directly interacting with the *dio3* promoter, but this effect seems to be insufficient to alter cortical TH action, as shown by the unaltered *luciferase* mRNA level.

## 3. Discussion

Environmental pollution is growing due to industrialization and intense arable and livestock farming. Many of the polluting compounds have been used widely for a long time, which has led to their accumulation in the environment [9]. As a consequence, chemical compounds reach the human population, and there is growing evidence suggesting that this interaction is associated with the development of human diseases [39,40,41]. Therefore, it has become critically important to uncover the potential hazards these molecules could exert on the population.

Endogenous, non-peptide hormones that control sexual functions, growth, cellular metabolism and differentiation often contain aromatic rings as a molecular backbone. Unsurprisingly, many cyclic and aromatic chemicals can interact with these molecules, thus interfering with various hormonal actions. Hormones of the thyroid gland represent no exceptions due to their aromatic amino acids that are critical for tissue function.

Impairments of TH action result in tissue disfunction, with documented consequences on human health [1,6]. TH economy is regulated by two major regulatory systems. Circulating TH levels are maintained centrally by the HPT axis, yet the target tissues have a striking autonomy to develop their own TH action by a cell-type specific local, intracellular regulatory system that involves TH activation and inactivation by deiodination, TH uptake by specific transporters and the TH receptor complex [4,5]. Due to this complex regulation, compounds like endocrine disruptors can affect TH economy also without affecting the circulating TH levels [42]. Therefore, it is of increasing interest to study the impact of endocrine disruptors on TH economy in a tissue-specific context.

In order to assess TH action in specific tissues of mice in the most unbiased way possible, we generated the THAI transgenic mouse model that expresses a specifically TH-responsive luciferase reporter system [15]. This model provides a proven approach to quantify the local TH action in microdissected brain regions, peripheral tissue samples and live mice by in vivo imaging [15,16,17]. Importantly, the assessment of TH signaling in this model is free from the confounding effects of non-TH-dependent pathways that are also able to impact the expression of endogenous TH-responsive genes, e.g., *enpp2*, a well-known endogenous TH marker gene that is also regulated by estrogen [43]. However, the reporter system of the THAI mouse overcomes these problems and provides a highly selective approach to assess TH action. While the THAI mouse provides a model to assess the disruptor potency of specific compounds in an intact mammalian tissue context, species-specific differences should be also kept in mind when extrapolating data. The picture could be further complicated by the species-specific metabolism of specific compounds, giving rise to bioactive metabolites. However, the THAI mouse can be subjected to in vivo imaging that allows to perform self-controlled experiments. This provides the advantage of studying the kinetics of the impact of disruptor exposure even during an extended time period that could much better model the real-life exposure of humans and animals. In addition, it also contributes to facilitating the reduction of the number of used experimental animals.

TBBPA and diclazuril treatments were performed according to literature data [27,44]. In comparison to the used dose, TBBPA concentration is significantly lower in human tissues (see Introduction), but it is obvious that only a fraction of the orally given dose will be deposited in tissues; furthermore, our exposure time was considerably shorter compared to real-life exposure.

TBBPA, a commonly used flame retardant, is known to impact TH economy. It was shown in an in vitro reporter assay in cultured neural cells that TBBPA can directly interfere with the response to T3 of TH-regulated genes [45]. It was also shown that TBBPA treatment decreased the circulating T4 levels in rats, which was accompanied with unchanged T3 and TSH levels [46].

We observed the same phenomenon in THAI mice. fT4 was decreased along with a strong trend toward an increase of TRH expression in the PVN that did not reach statistical significance. Based on this, it can be speculated that TBBPA acts at the level of the thyroid gland, e.g., by interfering with the hormone synthesis; the observed decrease in TH signaling in the bone and small intestine could be a direct consequence of decreased T4 levels. This hypothesis is supported by data showing that TH signaling in these tissues are markedly sensitive to the circulating TH levels compared to what observed in other tissues [15].

Tissue-specific TH action is a net result of TH availability and in certain tissues partially relies on the local deactivation of TH by the T3-degrading D3 enzyme. In *hdio3* promoter assays, we found that TBBPA stimulated the *dio3* promoter. This discovery suggests that altering the D3-encoding *dio3* gene activity can be another checkpoint where TBBPA potentially interferes with local TH action. This would represent a novel example of how endocrine disruptors act via altering hormone metabolism/inactivation, one of the categories listed by the recent Consensus Statement on the characteristics of endocrine-disrupting chemicals [47]. Generally, increased net *dio3* activity in the periphery could contribute to the observed lowered fT4 along with a relatively intact HPT axis. However, the tissue-specific changes of *dio3* expression point towards a more complex phenomenon, which makes it difficult to formulate generalized conclusions about the peripheral TH metabolism.

Importantly, the statistically not significant change in *trh* expression in the PVN does not exclude the possibility of the large observed difference being a functional response of the HPT axis. This seems to be controversial, since another study using hypothalamic in vivo transfection of a *trh-luc* construct into the mouse hypothalamus found either an increase or a decrease in *trh* transcription depending on the TBBPA treatment regime [27]. However, it is difficult to compare these findings with our data, since we measured endogenous *trh* expression in microdissected PVN samples in contrast to the mentioned study in which the transcriptional activity of a ~500-bp *trh* 5′FR-luc was assessed in the whole hypothalamus [27]. It can be speculated that the impact of TBBPA on the HPT axis is a net effect of various minor effects resulting in the partial downregulation of the global TH economy.

The veterinary drug diclazuril is used as a coccidiostatic agent [31,32]. Our data also provided evidence that in addition to TBBPA, diclazuril acts as a modulator of tissue-specific TH signaling. In contrast to TBBPA, all effects of diclazuril we observed in the THAI mice were stimulatory of TH action. Interestingly, these changes were accompanied with unchanged circulating TH levels and unchanged HPT axis parameters and a lack of a direct effect on the *dio3* promoter.

Considering that muscle, liver and fat have been reported to accumulate diclazuril [48], it is not surprising that the largest effects observed in the THAI mouse were found in these tissues. A longer exposure also allowed us to visualize the effect on BAT with in vivo imaging. Based on this, it is likely that the prolonged exposure increased the stimulatory capabilities of the drug on local TH action. However, after the recovery week, the observed effect disappeared, indicating a fast clearance in vivo. While these remarks suggest that diclazuril is rather an endocrine modulator than a disruptor, our data hint towards diclazuril being able to substantially modify the local TH action under continuous exposure.

In the case that the observed stimulatory effect of diclazuril occurred in treated poultry, this could result in elevated tissue energy expenditure and metabolism by increasing TH-dependent gene expression in the muscle and liver. While it has been thought that BAT-dependent thermogenesis is absent in the chicken, this has been recently questioned by demonstrating the emergence of beige-like fat as a physiological adaptation to cold [49]. This induction of BAT TH signaling by diclazuril could contribute to non-shivering thermogenesis and energy loss also in the chicken, but further studies are required to directly prove this hypothesis. Additionally, an effect of diclazuril on the growth performance and feed conversion of the chicken was studied, although the topic has not been widely investigated yet, and the results are controversial [50].

Medication may have a direct influence on growth performance, which might originate either from the direct modulation of tissue-specific TH signaling by a drug or from its inhibitory effect on subclinical or clinical coccidiosis, which of course per se negatively influences growth performance and body weight gain. In order to reveal a direct modulatory effect, studies on further target species are needed in the absence of coccidial infection. Would such effect truly exist, its connection to altered TH action would be plausible. 

In summary, our data provide evidence of a tissue specific disruption of TH signaling by TBBPA in the mouse, while also revealing the stimulatory effect of diclazuril on TH signaling without affecting the HPT axis. The current experiments also prove that THAI mouse can be used as an in vivo model to assess the potential of specific compounds to disrupt TH economy. In the BAT and small intestine, THAI mouse also provides a tool to perform self-controlled longitudinal studies on live mice to assess modulation of TH signaling.

## 4. Materials and Methods

### 4.1. Animals

The experiments were performed on ~90-day-old male THAI#4 mice; in vivo imaging experiments were performed on white furred THAI mice. Animals had food and water ad libitum and were housed under standard conditions. The experimental protocol was reviewed and approved by the Animal Welfare Committee at the Institute of Experimental Medicine (PE/EA/106-2/2021).

### 4.2. Animal Treatment and Sample Collection

TBBPA (Sigma) was delivered by oral gavage in corn oil containing 2% Et-OH as a saline suspension, in a dose of 150 mg/bwkg/day as described [27]. The treatment lasted 6 days, and control animals received the vehicle. Diclazuril (Sigma-Aldrich, St. Louis, MO, USA) was delivered by oral gavage in a dose of 10.0 mg/bwkg/day as a saline suspension as described [44]. Diclazuril treatment lasted 5 days, and control animals received the vehicle. Following the last treatment, the animals were sacrificed by decapitation, and trunk blood was collected. Peripheral tissues and brain regions were harvested and flash-frozen in dry ice. The PVN and ARC-ME regions were microdissected with the Palkovits punch technique; bone was collected from the distal part of the tibia and skeletal muscle was collected from musculus gastrocnemius. Treatment for in vivo imaging was continued for 14 days, followed by a 7-day-long withdrawal.

### 4.3. In Vivo Imaging

In vivo imaging was performed on anesthetized animals according to our established protocol, as previously described [15]. In short, THAI mice were anesthetized with ketamine–xylazine (50 and 10 μg/bwkg, respectively) i.p. Hair covering the abdominal or scapular regions was removed by a commercial depilatory cream, and D-luciferin (sodium salt, Gold Biotechnology, St. Louis, MO, USA) was introduced i.p. (150 µg/bwg). Images were taken after 15 min of incubation with 3 min acquisition time. Measurements were taken after 7, 14 and 21 days after the first day of treatment.

### 4.4. Serum Hormone Measurements

FT4 and fT3 levels were measured with the AccuLite CLIA Microwells kit (cat. no. 1275-300B and 1375-300B, respectively, Monobind Inc., Lake Forest, CA, USA) according to the manufacturer’s instructions in a Luminoskan Ascent (Thermo Fisher Scientific, Waltham, MA, USA) machine. However, TBBPA being a structural analogue, we were curious about whether it directly distorted the results. Spiked samples were used to elucidate this; control and treated animal sera were spiked with TBBPA in excess and resulted in the same concentrations as the unspiked samples. We concluded that the CLIA method was fit for our analytical purpose.

### 4.5. Taqman qPCR

Total RNA from tissues was isolated with the NucleoSpin RNA kit (Macherey-Nagel, Düren, Germany) according to the manufacturer’s instructions, with the following modifications. Non-brain samples were first homogenized with 1 mL Trizol reagent, extracted with 200 μL of chloroform and separated by centrifugation (15 min, 12,000× *g* on 4 °C). The supernatant was processed using the kit, as instructed. Then, 1 μg of total RNA was transcribed with the High-Capacity Reverse Transcription kit (Applied Biosystems, Waltham, MA, USA), as instructed. The product cDNA content was measured with the Qubit ssDNA assay (Invitrogen, Waltham, MA, USA), using 10 ng of cDNA in all Taqman reactions (Viia7, Applied Biosystems). The Taqman gene expressions assays are detailed in Table 1 (Thermo Fisher Scientific, Waltham, MA, USA). qPCR on microdissected brain regions of the THAI mouse was performed as described [17]. If a gene of interest was measured above 34 cycles, preamplification was performed with 5.55 ng of cDNA/reaction (Applied Biosystems). The preamplified DNA was not normalized for DNA content but only with respect to preamplified *hprt*. The details of qPCR are shown in the figure legends when relevant.

### 4.6. Cell Transfection and Luciferase Assay

The *hdio3* promoter–reporter construct contains 4327 bp of the 5′-flanking region plus 224 bp of the 5′-untranslated region of the human *dio3* gene. It was a gift of Prof. M. Dentice (University of Naples Federico II Italy) and was prepared as earlier described [51]. HEK293T cells were plated on 24-well plates in normal medium (89% DMEM, 10% FBS, 1% penicillin–streptomycin). Before transfection, the medium was changed to a hormone-free medium containing charcoaled FBS. The cells were transfected with the *dio3-luciferase* reporter, *Renilla luciferase* reporter and TRβ with X-tremeGene HP DNA transfection reagent (cat. no. 06366236001, Roche Basel, Switzerland) overnight. Then, the medium was replaced with hormone-free medium containing 50 nM T3 and/or 1 μM of TBBPA/diclazuril. The cells were harvested after 24 h of treatment. Luciferase and Renilla activity were measured with the Dual-Luciferase Reporter Assay System (Promega, Madison, WI, USA) according to the manufacturer’s instructions in a Luminoskan Ascent (Thermo Scientific, Waltham, MA, USA) machine as previously described [52].

### 4.7. Data Analysis

Data were analyzed with STATISTICA v13 software (Tibco Software, Palo Alto, CA, USA). Figures were prepared with Prism 9.3 (GraphPad Software Inc., San Diego, CA, USA). The figures show Tukey Box Plots; the box represents the two middle quartiles, the lower whisker represents the lower quartile, the upper whisker represents the upper quartile, the line represents the median, the dots represent outlier data. The number of used animals is indicated in figure legends. Null-hypothesis significance tests were conducted with a 95% level of confidence. The Student’s two sample two-sided t test was used to analyze two groups; one-way analysis of variance (ANOVA) followed by Tukey post-hoc test was used to compare more than two groups; ANOVA was applied as within-subjects ANOVA for in vivo imaging data. The models were deemed adequate based on residual plots and residual normal plots.

## Figures and Tables

**Table 1:** Taqman gene expression assays.

Gene Symbol	Gene Name	Assay ID
*actinb*	β actin	Mm02619580_g1
*dCpG luciferase*	dCpG luciferase reporter (custom made)	AIY9ZTZ
*dio3*	deiodinase, iodothyronine type III	Mm00548953_s1
*gapdh*	glyceraldehyde-3-phosphate dehydrogenase	Mm99999915_g1
*hprt1*	hypoxanthine guanine phosphoribosyl transferase	Mm01545399_m1
*slc16a2*	MCT8, monocarboxylate transporter 8	Mm01232724_m1
*trh*	Thyrotropin releasing hormone	Mm01963590_s1
*tshb*	thyroid stimulating hormone, beta subunit	Mm03990915_g1

## Data Availability

Data are available on request.
